# Cytomorphological Spectrum of Hepatic Lesions on Ultrasonography (USG) or CT-Guided Fine-Needle Aspiration Cytology (FNAC) in a Tertiary Care Centre, Rajendra Institute of Medical Sciences (RIMS), Ranchi, Jharkhand, India

**DOI:** 10.7759/cureus.70269

**Published:** 2024-09-26

**Authors:** Smita Kumari Gupta, Tripti Ashu, Monika Bharti, Manoj K Paswan, V S Sindhu, Venkatesh N

**Affiliations:** 1 Pathology, Rajendra Institute of Medical Sciences, Ranchi, IND; 2 Pathology, Sheikh Bhikhari Medical College, Hazaribagh, IND; 3 Community Medicine, Rajendra Institute of Medical Sciences, Ranchi, IND

**Keywords:** cholangiocarcinoma, core needle biopsy, cystic lesion, cytomorphology, hepatic lesion, hepatoblastoma, hepatocellular carcinoma, metastatic lesion, usg-guided fnac

## Abstract

Background

Ultrasonography (USG)-guided fine-needle aspiration cytology (FNAC) of the liver is a primary diagnostic procedure for primary and metastatic hepatic lesions. Despite histopathology being the gold standard, the outcomes of ultrasound-guided fine needle aspiration cytology are encouraging.

Aims and objective

The purpose of the study is to determine the diagnostic utility of ultrasonography (USG) or computerized tomography (CT)-guided FNAC in the detection of liver lesions, to investigate cytomorphological patterns of liver lesions identified by guided FNAC, and, wherever feasible, to correlate FNAC diagnosis with histopathology and imaging modalities.

Materials and methods

This was a hospital-based observational study of 62 patients carried out in the Department of Pathology, Rajendra Institute of Medical Sciences (RIMS) Ranchi during a period of 1.5 years from August 2020 to February 2022. The patients with suspected hepatic lesions were subjected to ultrasound-guided or CT-guided fine needle aspiration cytology (FNAC) following clinical and radiological evaluation and cytomorphological features were analyzed.

Results

Cyto-morphological diagnosis of 62 cases was categorized into 19 (30.64%) non-neoplastic lesions and 43 (69.35%) malignant neoplastic lesions. The different neoplastic lesions were 17 (27.41%) hepatocellular carcinoma, 1 (1.61%) hepatoblastoma, 1 (1.61%) cholangiocarcinoma, 23 (37.09%) metastatic adenocarcinoma and one case (1.61%) of unclassified malignancy. Histopathological correlation for confirming the diagnosis could be done in 33 malignant neoplastic lesions and the concordance rate of FNAC with respect to histopathological examination (HPE) was 92.11%. Overall diagnostic accuracy of the FNAC of liver to detect malignant lesions was 98.39%.

Conclusion

Compared to ultrasonography alone, ultrasound or CT-guided fine needle aspiration of the liver has a more promising role in the diagnosis and classification of hepatic lesions as it demands a higher level of precision to achieve diagnostic accuracy.

## Introduction

Carcinoma of the liver, whether primary or metastatic, usually has a terrible prognosis because it is usually incurable at the time of diagnosis [1]. A diagnostic technique like fine-needle aspiration cytology (FNAC) should be taken into consideration early in the investigative process as it provides accuracy without complications and requires little intervention at a reasonable cost. Its benefits include a far lower chance of complications and much less discomfort, which helps to prevent hospital stay. Single or multiple focal abnormalities detected by palpation, computed tomography (CT) or ultrasonography (USG) constitute the main indications for FNAC of the liver [1]. Primary liver tumors, benign or malignant metastatic deposits, congenital and acquired cysts, abscesses, and granulomas are among the differential diagnoses for hepatic mass lesions. FNAC is becoming more and more common as a diagnostic method for space-occupying liver lesions since it is less invasive, quicker, and less expensive than core-needle or open biopsy [2,3]. Blind aspiration does, however, have the intrinsic disadvantage of having poorer diagnostic accuracy and poor lesion localization. Due to this, other radiologically guided FNAC techniques are now being used. These techniques involve the use of either computerized tomography (CT) or ultrasonography (USG). Enormous progress has been achieved in cross-sectional imaging methods such as computed tomography, ultrasonography, and magnetic resonance imaging [1].

Despite these improvements, imaging is diagnostic in only a few pathological conditions of the liver. These imaging modalities are very reliable in distinguishing a solid mass from a cystic one, but at times fail to detect the mother tissue properly and to state whether the lesion is benign or malignant. So the need for a more accurate investigation that can correctly state the nature of diseased tissue arises. Tissue sampling is needed for diagnosing the majority of pathologies which would subsequently determine their management. FNAC served this purpose very well. So the concept of supplementing FNAC with USG or CT guidance was introduced.

Image-guided procedures have increased the safety and accuracy of percutaneously performed FNACs resulting in greater utilization of these procedures. USG/CT-guided FNAC offers a better approach to diseased tissue whereas FNAC can be done avoiding the vital structures of the abdomen. Previously inaccessible lesions can be safely sampled [4]. It has led to a reduction in open biopsies and two-stage surgical procedures by providing a definitive diagnosis prior to primary surgical treatment [4]. With this background, the present study was conducted with the purpose of diagnosing focal hepatic lesions with minimal injury, time, and complications, and to determine the diagnostic efficacy of guided FNAC in neoplastic and non-neoplastic lesions of the liver.

## Materials and methods

**Figure 1:**
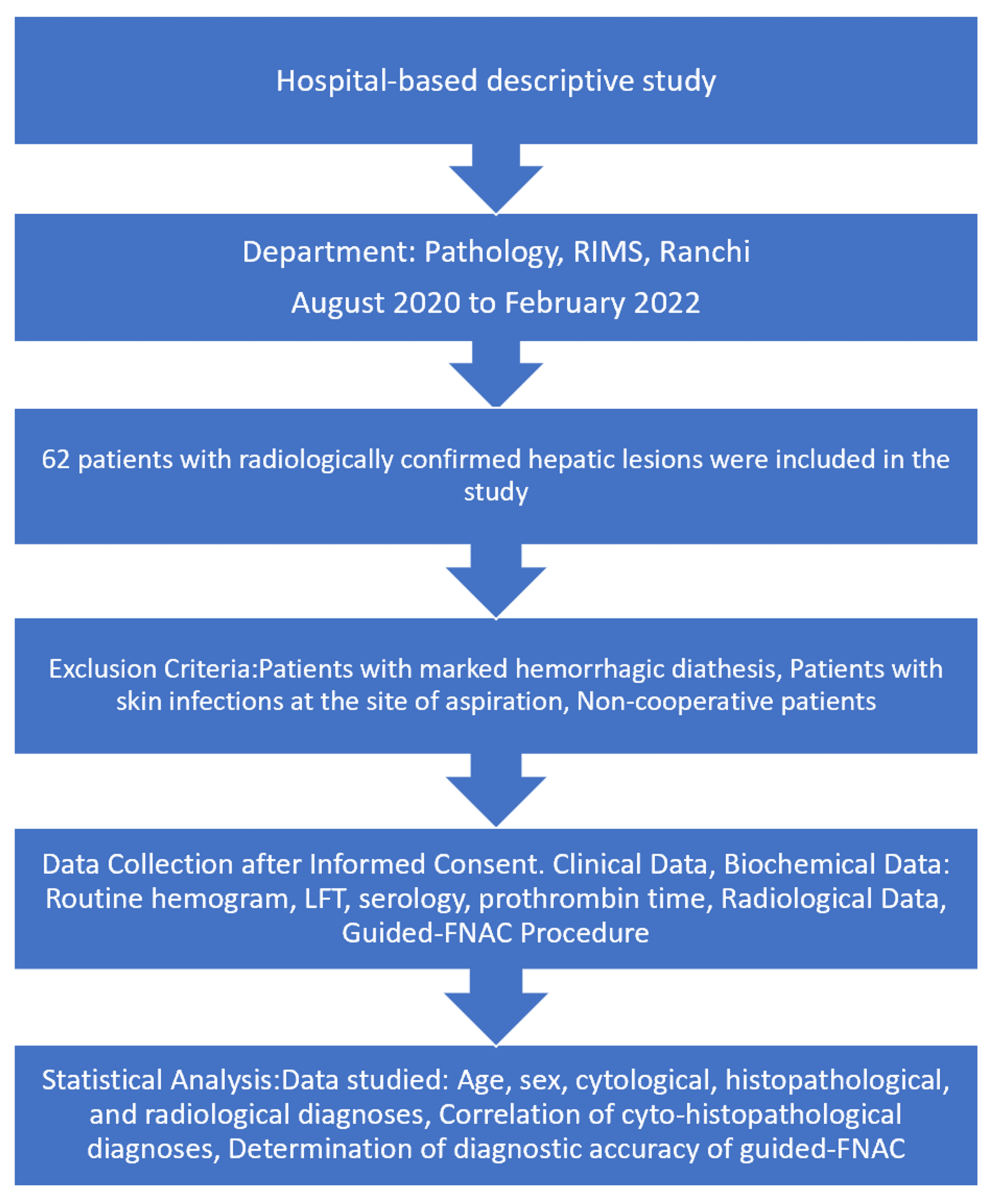
Overall flow of the study (n=62)

This was a hospital-based descriptive study conducted on 62 patients with radiologically confirmed hepatic lesions during the period of 1.5 years from August 2020 to February 2022 in the Department of Pathology, Rajendra Institute of Medical Sciences (RIMS), Ranchi. The exclusion criteria for this study included patients with hepatic masses confirmed by radiological examination, those with marked hemorrhagic diathesis, patients with skin infections at the site of aspiration, and non-cooperative patients. Detailed clinical, biochemical (routine hemogram, liver function test [LFT], serology and prothrombin time) and radiological data along with the patient’s informed consent was obtained. Patients with normal prothrombin time were subjected to guided FNAC. The area (based on the clinical examination and radiological findings) was sterilized with spirit. 22-gauge, 90 mm disposable lumbar puncture needle with trocar was placed against the skin at the predetermined puncture site and inserted into the lesion under USG/CT guidance with a single quick motion after confirming the position of the lesion. Once the needle was in the lesion, the trocar was removed and a well-fitting 10 ml or 20 ml disposable plastic syringe was attached to the needle. The cytological material was aspirated by creating negative pressure and the smears were prepared immediately. Two dry smears and two smears fixed in alcohol 95% were prepared. Alcohol-fixed smears were stained with Papanicolaou (PAP)/ hematoxylin and eosin (H&E) and dry slides were stained with Leishman’s stain. The HPE specimen (38/62) was obtained by core needle biopsy done during aspiration. Statistical data of age, sex, cytological diagnosis, histopathological diagnosis, and radiological diagnosis was studied. Cyto-histopathological diagnoses were correlated and the diagnostic accuracy of guided FNAC was determined. The overall methodology is given in Figure 1.

## Results

Frequency distribution of cases by age (n=62)

Over two years, 62 patients with hepatic lesions underwent ultrasound (USG) or computed tomography (CT)-guided fine-needle aspiration cytology (FNAC). Of these patients, 33 (53.23%) were males, and 29 (46.77%) were females. The distribution of age and sex in the study group is detailed in Table 1. The majority of patients fell within the 51-60 years age group, comprising 30.65% of the study population. This was followed by the 31-40 years age group, which accounted for 20.96% of the patients. Twelve patients (19.35%) were between 41-50 years old, and 10 patients (16.13%) were in the 61-70 years age group. Overall, 87% of the patients were aged between 31 and 70 years.

**Table 1:** Distribution of cases with respect to age

Age in years	Male	Female	Total
< 10	1	0	1
11-20	2	1	3
21-30	1	1	2
31-40	8	5	13
41-50	3	9	12
51-60	11	8	19
61-70	6	4	10
71 & above	1	1	2
Total	33	29	62

Results of USG/CT-guided FNAC (n=62)

Out of the 62 patients, 43 cases (69.35%) were found to be malignant based on the guided FNAC results. Additionally, 13 cases (20.96%) were identified as inflammatory lesions, two cases (3.22%) as benign cystic lesions, and four cases (6.45%) were diagnosed as regenerative nodules. Among the 43 malignant lesions, 19 (44.19%) were primary malignancies, while 23 (53.49%) were metastatic as shown in Table 2. One case (2.33%) could not be classified as either primary or metastatic based on the guided FNAC.

**Table 2:** Results of guided FNAC FNAC: Fine-needle aspiration cytology; HCC: Hepatocellular carcinoma

Diagnosis	Total	Frequency
NEOPLASTIC	42	67.74%
PRIMARY MALIGNANCY	19	30.64%
HCC	17	27.42%
Hepatoblastoma	1	1.61%
Cholangiocarcinoma	1	1.61%
METASTATIC LESION	23	37.10%
Metastatic adenocarcinoma	20	32.25%
Metastatic squamous cell carcinoma	1	1.61%
Metastatic small cell carcinoma	2	3.22%
NON-NEOPLASTIC	19	30.65%
Pyogenic Liver Abscess	11	17.74%
Amoebic Liver Abscess	2	3.22%
Benign Cystic Lesion	02	3.22%
Regenerative Nodule	04	6.45%
UNCLASSIFIED MALIGNANCY	01	1.61%
TOTAL	62	100

Distribution of primary liver malignancies and liver metastasis (n=23)

Nineteen cases of primary liver malignancies included 17 cases of hepatocellular carcinoma, one case of hepatoblastoma, and one case of cholangiocarcinoma. The details of the primaries in case of metastatic lesions are provided in Table 3.

**Table 3:** Primary sites for liver metastasis

Metastasis	No. of cases
Metastatic-Large Bowel	14
Metastatic-Gallbladder	3
Metastatic-Lungs	2
Metastatic-Breast	1
Adenocarcinoma With Unknown Primary	3

Cyto-histopathological correlation

In 38 cases, the cytological diagnosis was correlated with the core needle biopsy diagnosis. The histopathological examination (HPE) diagnosis was considered the gold standard for comparison (Table 4). The concordance rate between FNAC and HPE diagnoses was 92.11%.

**Table 4:** Cyto-histopathological correlation

Cytological Diagnosis	Core Needle Biopsy	Histological Diagnosis	
		Non-Malignant (No. of cases)	Malignant (No. of cases)
Malignant (43)	33	0	33
Non-Malignant (19)	4	3	1
Suspicious of Carcinoma (1)	1	0	1

Correlation of ultrasound/CT findings with USG/CT-guided FNAC findings

Ultrasonographic and CT findings of the liver were correlated with the cytological findings obtained from USG-guided FNAC. Table 5 shows a strong correlation between specific imaging findings and their corresponding cytological diagnoses. Hepatocellular carcinoma (HCC) was the most frequently diagnosed malignancy, often associated with single or multiple masses on imaging. Metastatic lesions were also common, typically appearing as multiple target-like lesions.

**Table 5:** Radiological correlation SOL: Space occupying lesion HCC: Hepatocellular carcinoma

USG Findings/CT Findings	Cytological Findings	Frequency
Single mass	HCC	7
Multiple masses	HCC	4
Large single isodense mass	HCC	4
Multiple hypodense mass	HCC	2
Unifocal, heterogeneously echogenic mass in the right lobe of the liver with calcification and area of necrosis	HEPATOBLASTOMA	1
Enhancing mass in the right lobe of the liver with dilatation of intrahepatic biliary radicles	CHOLANGIOCARCINOMA	1
Strongly echogenic SOL in the right lobe	UNCLASSIFIED MALIGNANCY	1
Single round to oval anechoic mass in the right lobe of the liver with posterior acoustic enhancement	BENIGN CYSTIC LESION	2
Multiple well-defined hypoechoic areas in the liver with central debris	INFLAMMATORY LESION	8
Single hypoechoic lesion	INFLAMMATORY LESION	2
Heterogeneously hypoechoic lesion with shaggy margins	INFLAMMATORY LESION	1
Multiple ring-enhancing hypodense lesions	INFLAMMATORY LESION	1
Peripherally enhancing hypodense lesion in the right lobe with thick wall	INFLAMMATORY LESION	1
Well-defined isoechoic nodules of various sizes	REGENERATIVE NODULE	3
Well-defined isodense nodules	REGENERATIVE NODULE	1
Revealed multiple enhancing masses of various sizes which were lower in attenuation than surrounding liver parenchyma	METASTATIC LESION	3
Multiple rounded target-like lesions (well-defined areas of altered echogenicity surrounded by a sharply defined poorly echogenic halo	METASTATIC LESION	18
Single target like lesion	METASTATIC LESION	2

Comparison of guided FNAC results with clinico-radiological diagnosis

The results of guided FNAC were compared with clinico-radiological assessments. Of the 62 cases, 42 (67.74%) were clinically and radiologically diagnosed as malignant lesions, while 20 (32.26%) were identified as nonmalignant. Among the 42 cases clinically and radiologically diagnosed as malignant, 41 were confirmed as malignant by guided FNAC, with one case found to be nonmalignant (this case involved multiple hepatic abscesses initially diagnosed as metastatic deposits based on clinico-radiological findings). Of the 20 cases clinically and radiologically diagnosed as nonmalignant, 18 were confirmed as nonmalignant on guided FNAC, while two cases were found to be malignant. These two cases were hepatocellular carcinomas that were missed on CT imaging due to the presence of a cirrhotic background. This is shown in Table 6.

**Table 6:** Comparison of cytological diagnosis with clinico-radiological diagnosis

Clinico-radiological Diagnosis	No. of cases	Cytological Diagnosis	
		Malignant	Nonmalignant
Malignant	42	41 (97.62%)	1 (2.38%)
Nonmalignant	20	2 (10.0%)	18 (90.0%)

In all cases included in this study, the final diagnosis was obtained through clinical features supported by various investigations, histopathological reports (where available), and/or responses to treatment with the clinical course of the disease.

Diagnostic performance of guided FNAC

**Figure 2:**
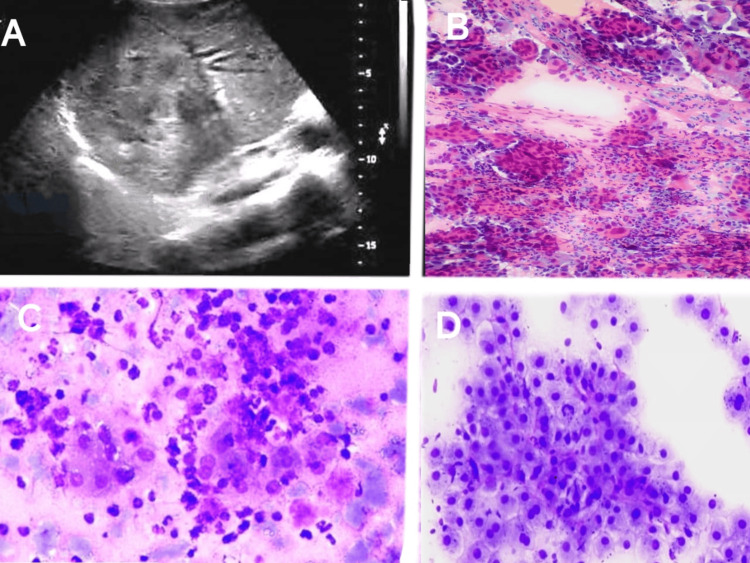
USG & guided FNAC pictures of hepatic lesions A - Ultrasonogram of the liver mass with heterogenous echotexture B - Guided FNAC of the liver showing cholangiocarcinoma C - Guided FNAC of the liver showing pyogenic liver abscess D - Guided FNAC of the liver showing hepatic regenerative nodule USG: Ultrasonography; FNAC: Fine-needle aspiration cytology

**Figure 3:**
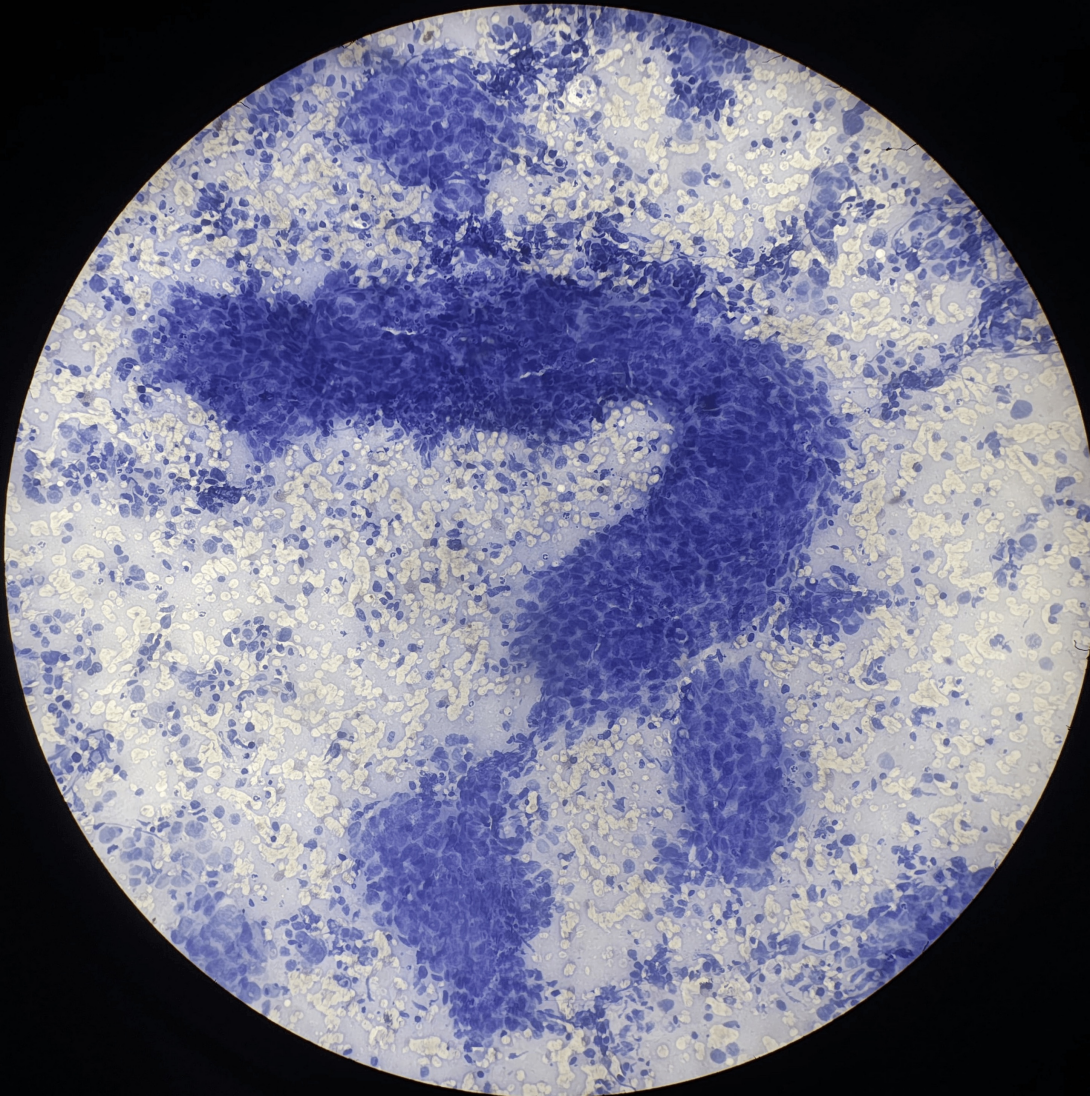
USG-guided FNAC showing thick trabeculae of cells in HCC USG: Ultrasonography; FNAC: Fine-needle aspiration cytology; HCC: Hepatocellular carcinoma

**Figure 4:**
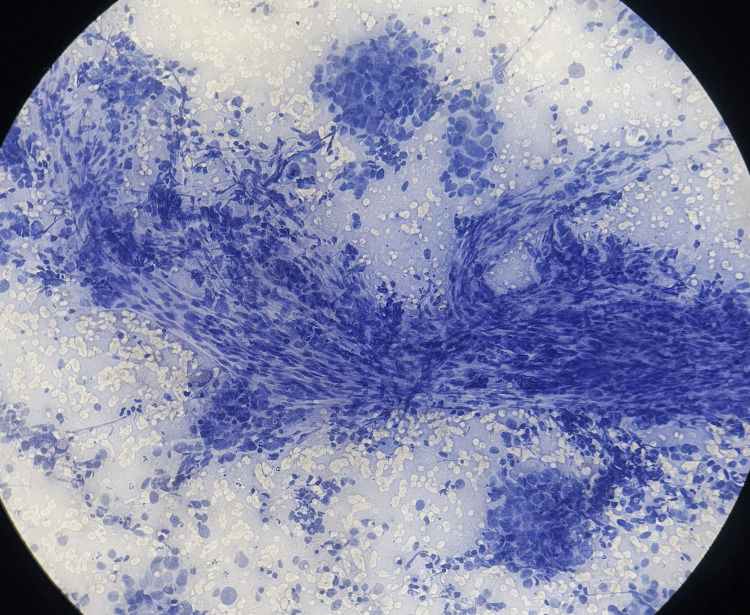
USG-guided FNAC of HCC showing transgressing of capillaries. USG: Ultrasonography; FNAC: Fine-needle aspiration cytology; HCC: Hepatocellular carcinoma

**Figure 5:**
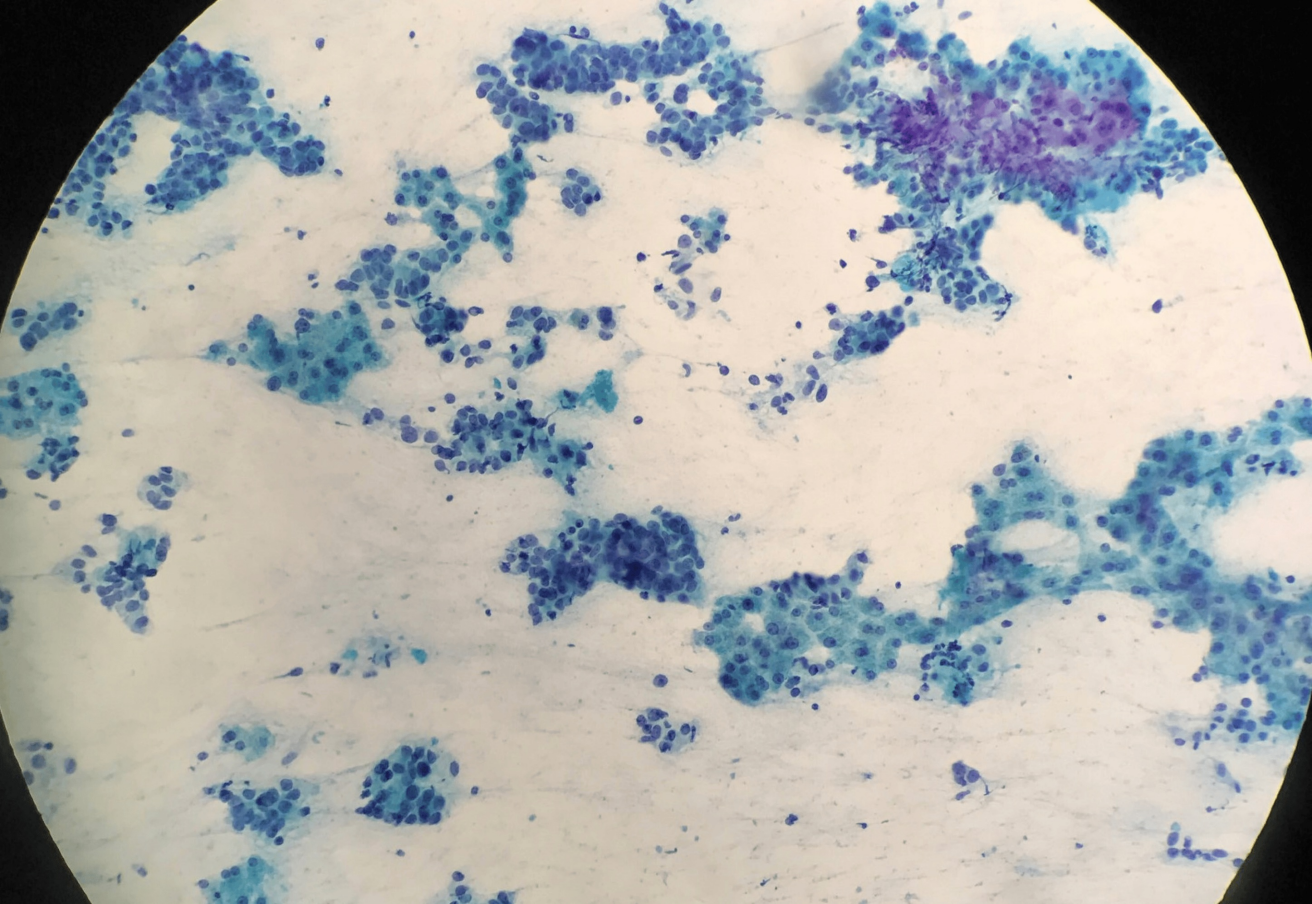
Guided FNAC of liver showing metastatic adenocarcinoma from breast. FNAC: Fine-needle aspiration cytology

**Figure 6:**
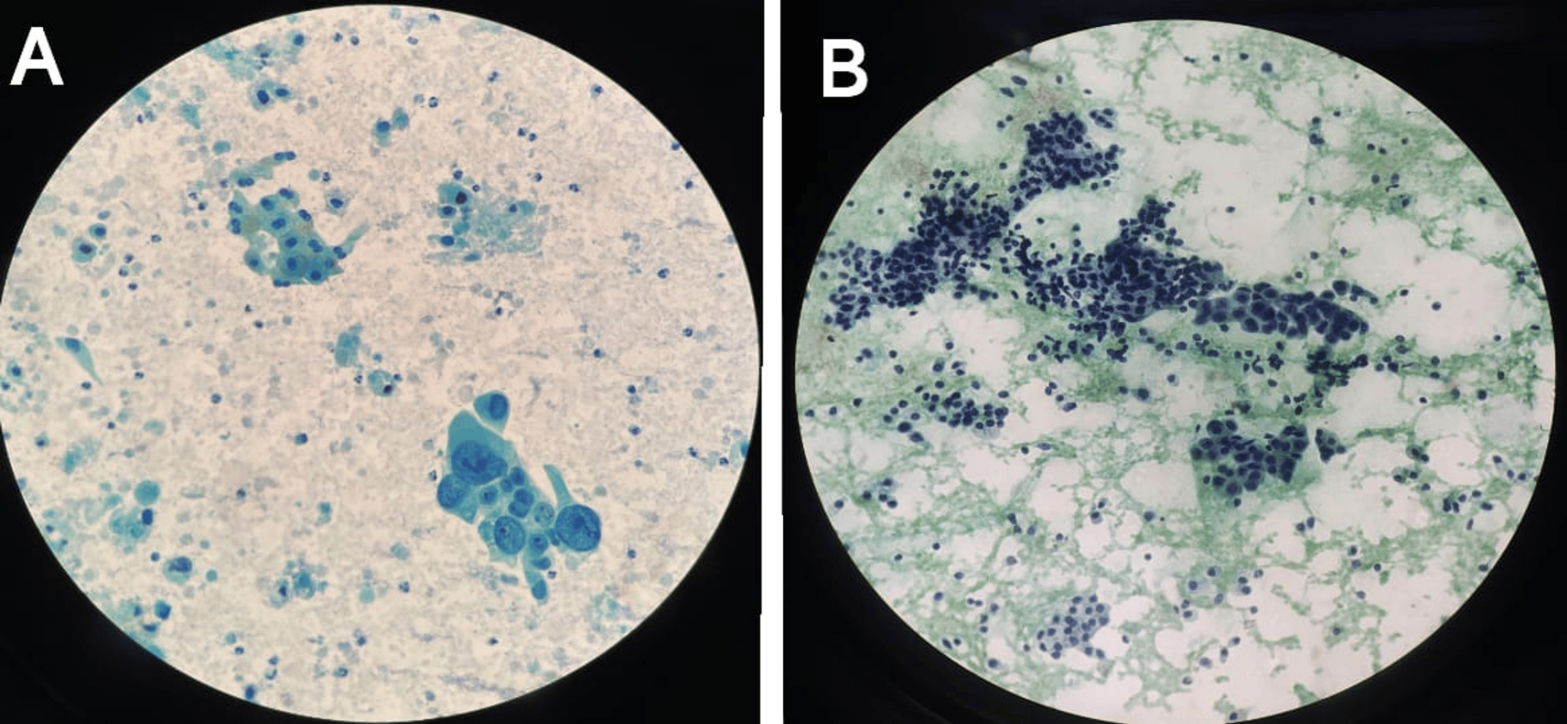
Guided FNAC of liver showing metastasis from lungs (A) and gallbladder (B) A - Metastatic adenocarcinoma from lungs. B - Metastatic adenocarcinoma from gallbladder (cluster of tumor cells admixed with normal hepatocytes). FNAC: Fine-needle aspiration cytology

**Figure 7:**
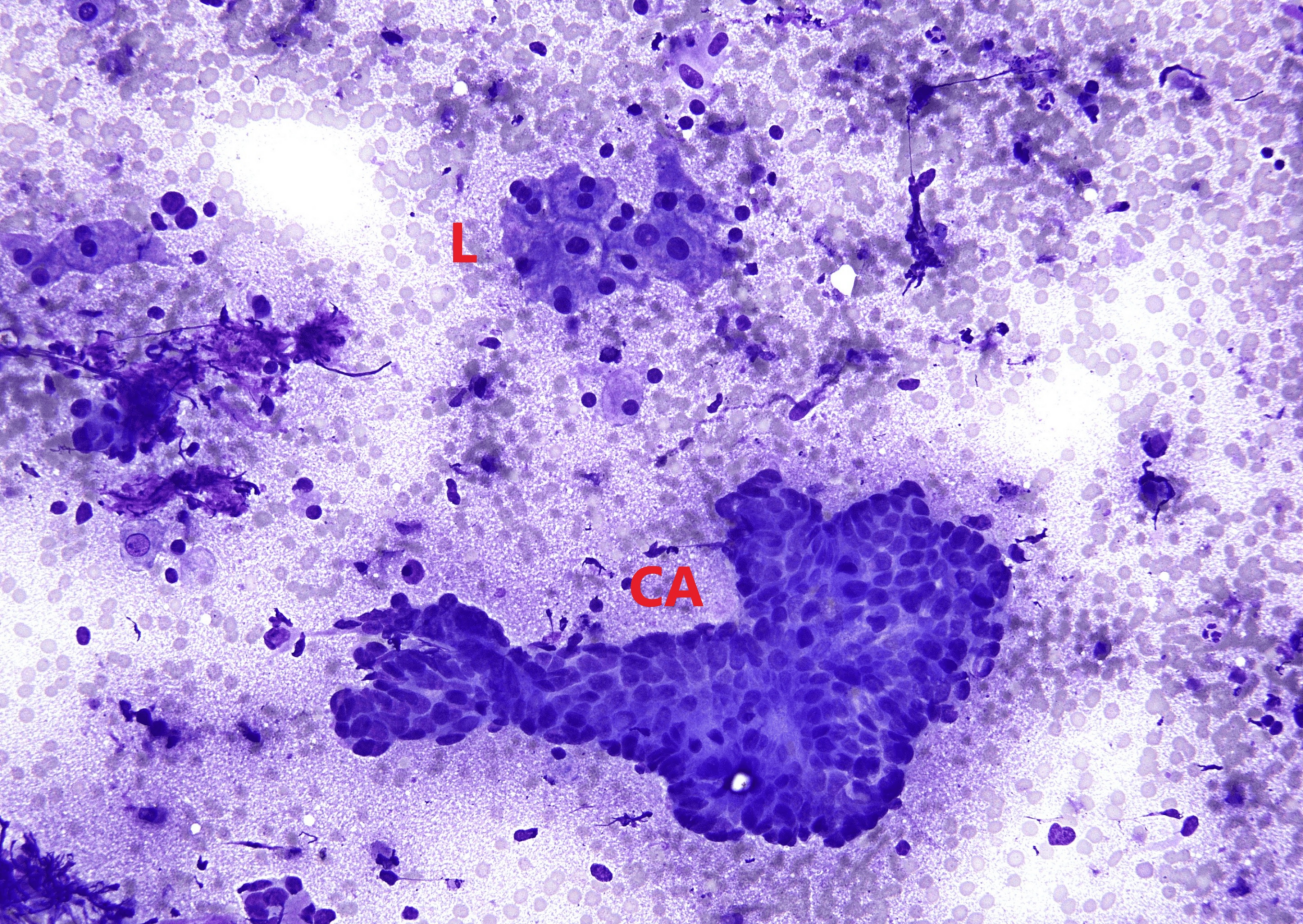
Guided FNAC of liver showing metastatic adenocarcinoma from colon L--> Liver (Hepatocytes) CA--> Carcinoma (Tumor cells) FNAC: Fine-needle aspiration cytology

**Figure 8:**
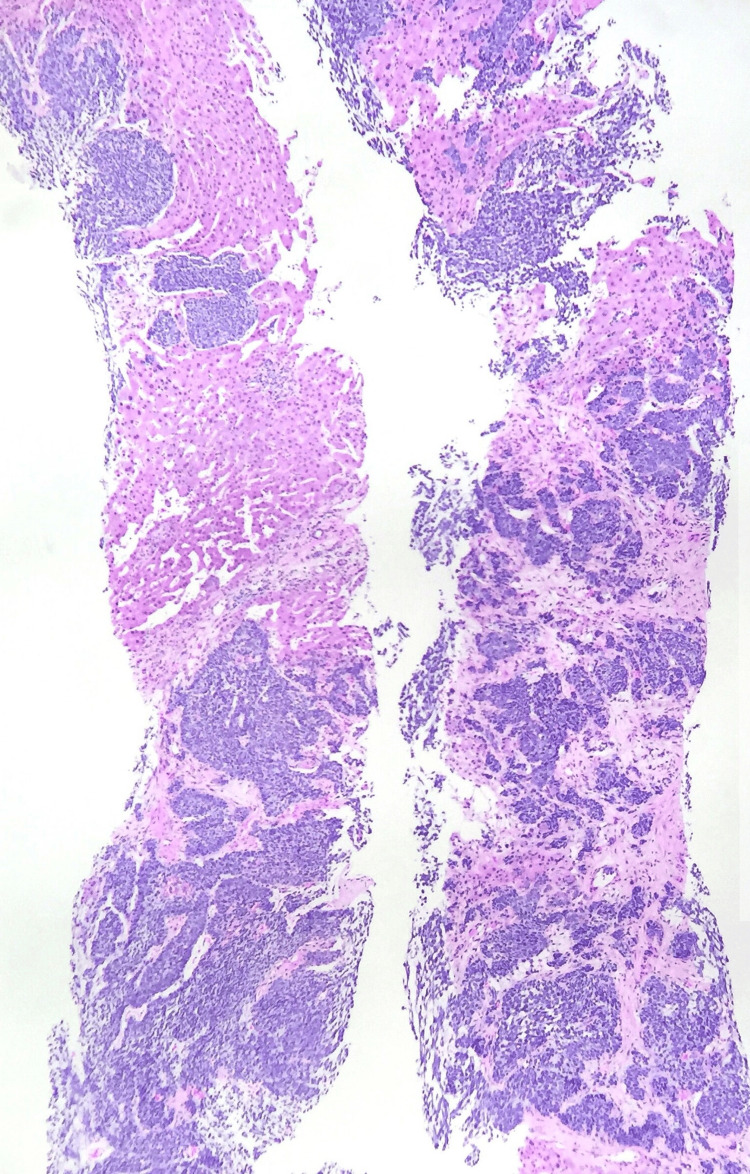
Trucut biopsy of liver showing metastatic small cell carcinoma from lungs

**Figure 9:**
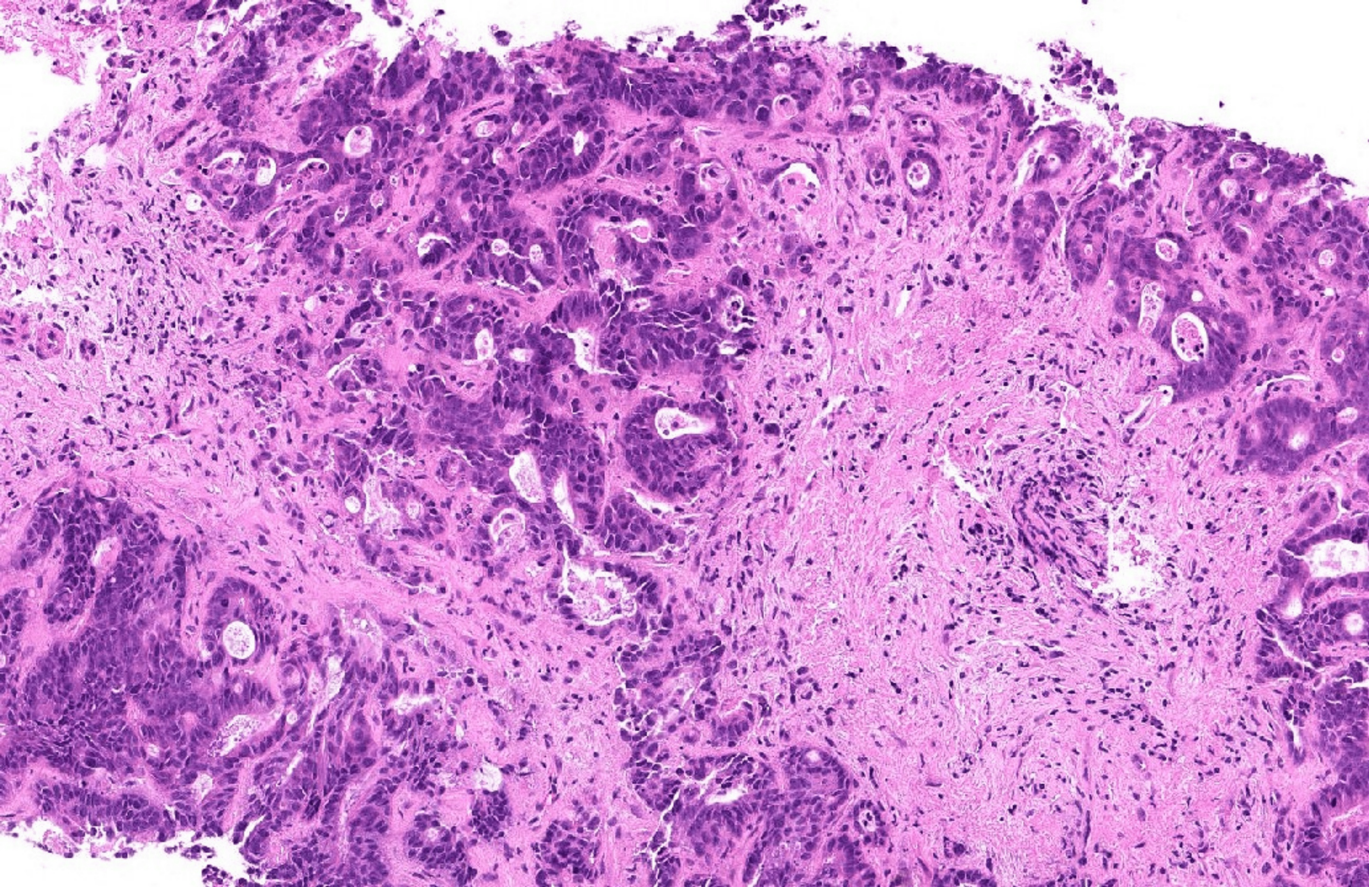
Biopsy of liver showing metastatic adenocarcinoma

**Figure 10:**
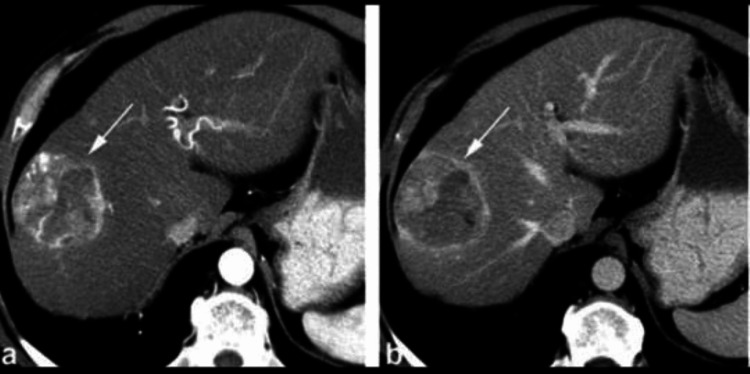
Radiograph showing primary liver lesion

**Figure 11:**
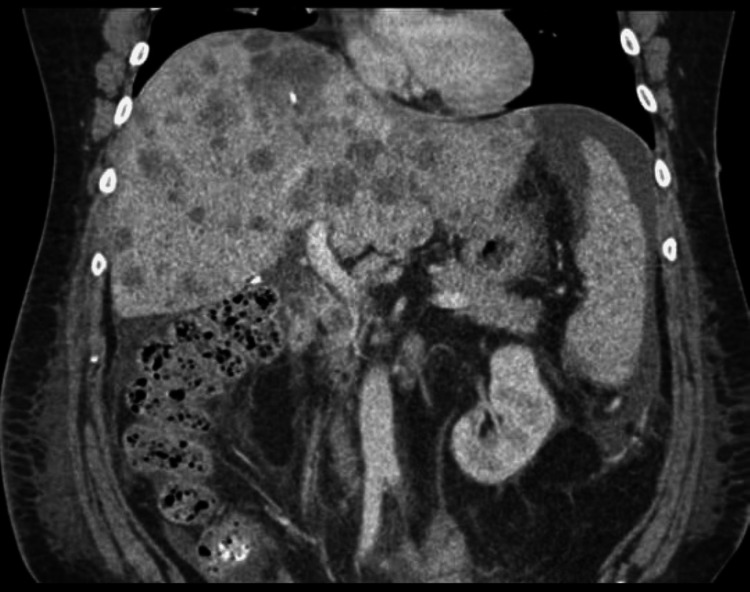
Radiograph showing liver metastasis

The diagnostic performance of guided FNAC for detecting malignancy in hepatic lesions was highly effective, with a sensitivity of 97.73% and a specificity of 100%. The procedure demonstrated a positive predictive value (PPV) of 100%, indicating that all patients diagnosed with malignancy via FNAC were confirmed to have malignant lesions. The negative predictive value (NPV) was 94.74%, reflecting the accuracy in ruling out malignancy in non-malignant cases. The overall accuracy of guided FNAC in this study was 98.39%, with a prevalence of malignancy among the population of radiologically confirmed hepatic lesions at 70.97% indicating the reliability of guided FNAC as a diagnostic tool for malignancy in hepatic lesions. The radiology (USG and CT), cytology and histopathology (hematoxylin and eosin) images of different hepatic lesions are shown in Figures 2-11.

## Discussion

In this study, guided FNAC was performed in 62 patients with hepatic lesions. There were 33 (53.23%) males and 29 (46.77%) females. The age range of patients was 3 to 75 years. Samples were adequate in 60 (96.77%) patients. Two (3.23%) patients had to be examined twice to obtain a sufficient sample. Inadequate sampling rate of 3.23% in the first attempt is comparable to those reported by Gallegos et al. (2.5%) [5] and Talukder et al. (6.5%) [6]. This was safely reduced by repeat aspiration under USG/CT guidance. Of 62 patients, 43 (69.35%) cases were interpreted as malignant lesions and 19 (30.65%) cases as nonmalignant lesions. This is comparable to observations made by Gallegos et al. [5] and Herszenyi et al. [7].

Among malignant lesions, metastatic deposits were the commonest (53.49%). This is consistent with observations made by Talukder et al. [6] and Gatphoh et al. [8]. The commonest primary site for metastatic lesions was large bowel in 14 (60.87%) cases. This is comparable to observations made by Ohlsson et al. [9], and Tot and Samii [10]. Gallbladder was the primary site in three cases (13.04%), lungs in two (8.69%) and breast in one (4.35%), and the primary site was unknown in three (13.04%) cases. Three cases of metastatic lesion with unknown primary were later subjected to needle biopsy. Two cases were proved to be metastatic adenocarcinoma while one case came out to be metastatic small cell carcinoma. Thus adenocarcinomas comprised 19 (82.60%) cases of metastatic lesions.

The overall diagnostic accuracy of FNAC was 98.39% which was concordant with the study by Swamy et al. where the overall diagnostic accuracy of FNAC was 97.82% with a sensitivity and specificity of 96.87% and 100%, respectively [11]. The sensitivity, specificity and diagnostic accuracy of guided FNAC for malignancy were 97.73%, 100% and 98.39% respectively with a positive predictive value of 100% and a negative predictive value of 94.74%. Guo et al. [12] reported the sensitivity and specificity of FNAC for malignancy as 95.1% and 100% with a positive predictive value of 100% and negative predictive value of 88.8% which are almost comparable to observations of this study. Soyuer et al. [13] also reported the sensitivity and specificity of the procedure as 99.5% and 100% respectively. Similar results were seen in the study by Namshiker et al. where the overall diagnostic accuracy of FNAC was 90% and the diagnostic sensitivity for malignant hepatic lesions was 98% with a positive predictive value of 94% [14].

There was no false positive diagnosis in this study. This is consistent with observations made by Zhang et al. [15] and Gallegos et al. [5]. A striking finding in this study was the total absence of any complications. This is comparable to the findings of Xu [16] and Al-Damegh [17]. However, chances of complications like haemorrhage, bile leak needle track seeding, etc. are more with a wide-bore needle biopsy. Many complications of histologic biopsy are obviously the result of puncture trauma related to the outer diameter of the needle and the use of a fine needle greatly reduces this hazard [18]. This is very much true for this study where the use of 22G needles did not lead to any complications.

According to the study by Rasania et al., USG-guided FNA was very helpful in making cytological diagnosis of hepatic masses in 90% of cases which is similar to our study (92.11%) [19]. The main drawback of the study is the small sample size. The result of the study has limited generalizability for the tertiary centers of the whole country with different population sizes and caseloads. Hence the study could have been better if studied in a larger population.

## Conclusions

This study emphasizes the high diagnostic accuracy of ultrasound (USG) and computed tomography (CT)-guided fine-needle aspiration cytology (FNAC) in identifying hepatic lesions. FNAC proves to be an essential tool for diagnosing liver lesions including both primary and metastatic malignancies with a diagnostic accuracy of 98.39%. The guided FNAC technique offers a minimally invasive, cost-effective, and highly precise diagnostic method, reducing the need for more invasive procedures like open biopsies and enabling timely and accurate clinical management of hepatic diseases. This approach has also demonstrated an excellent safety profile, with no reported complications, making it a preferable choice in clinical settings. The integration of imaging and cytological evaluation provides a comprehensive approach to diagnosing liver lesions, ensuring accurate and timely clinical decision-making. A multidisciplinary approach, incorporating imaging, cytology, and clinical findings, is essential for accurate diagnosis and management of liver lesions. Future studies with larger sample size and longer follow-up periods are recommended to further validate these findings and explore the potential of guided FNAC in the management of hepatic lesions.

## Figures and Tables

**Figure 12:**
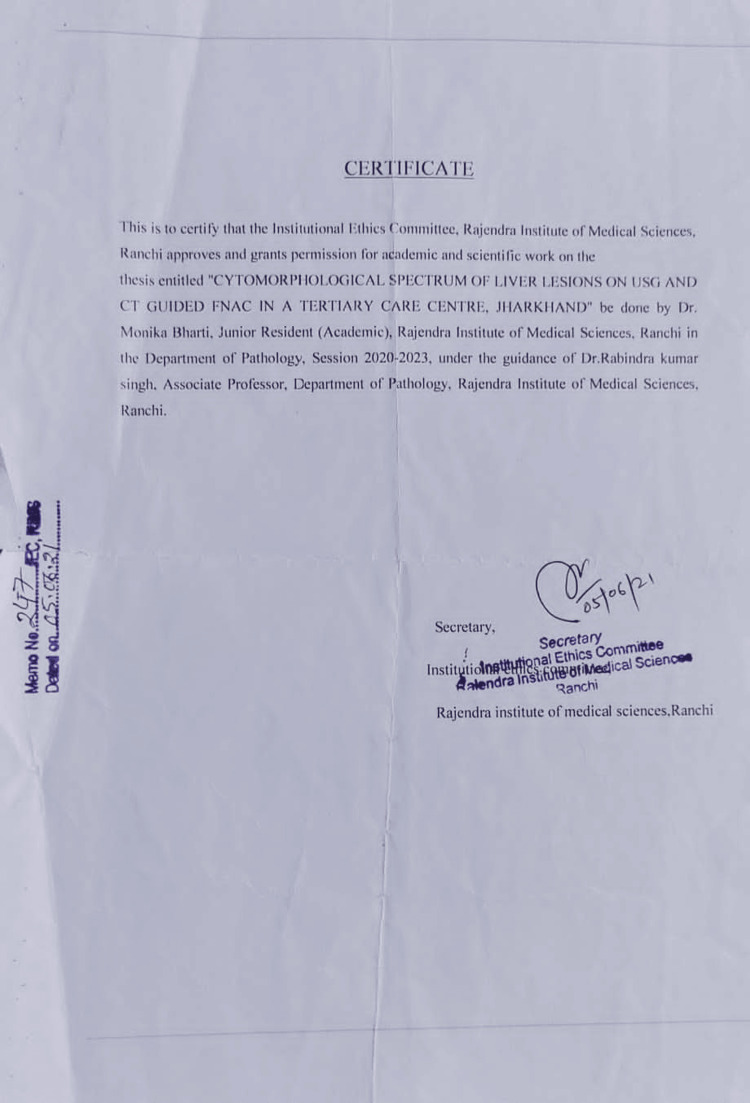
IEC approval letter

## References

[1] Lekha MB, Prabhu DC, Nagarjappa AH (2018). Ultrasound/computerized tomography guided fine needle aspiration cytology of liver lesions. IP J Diagn Pathol Oncol.

[2] Dhameja N, Rai V, Singh R (2016). Fine needle aspiration cytology of liver space occupying lesions - A study of 57 cases from a tertiary care centre. Int J Sci Res (IJSR).

[3] Rajyalakshmi R, Rajani V (2020). Ultrasound guided FNAC of focal lesions of liver. Indian J Pathol Oncol.

[4] National Guideline Centre (UK) (2019). Imaging for Fine Needle Aspiration: Thyroid Disease: Assessment and Management: Evidence Review N. National Institute for Health and Care Excellence (NICE).

[5] Gallegos M, Cruz F, Quintana ME, Duarte I (1997). Cytodiagnosis of focal hepatic lesions by image guided fine needle biopsy [Article in Spanish]. Rev Med Chil.

[6] Talukder SI, Huq MH, Haque MA (2004). Ultrasound guided fine needle aspiration cytology for diagnosis of mass lesions of liver. Mymensingh Med J.

[7] Herszényi L, Farinati F, Cecchetto A (1995). Ultrasound guided fine-needle aspiration biopsy in the diagnosis of hepatocellular carcinoma [Article in Hungarian]. Orv Hetil.

[8] Gatphoh ED, Gaytri S, Babina S, Singh AM (2003). Fine needle aspiration cytology of liver: a study of 202 cases. Indian J Med Sci.

[9] Ohlsson B, Nilsson J, Stenram U, Åkerman M, Tranberg KG (2002). Percutaneous fine-needle aspiration cytology in the diagnosis and management of liver tumours. Br J Surg.

[10] Tot T, Samii S (2003). The clinical relevance of cytokeratin phenotyping in needle biopsy of liver metastasis. APMIS.

[11] Swamy M, Arathi C, Kodandaswamy C (2011). Value of ultrasonography-guided fine needle aspiration cytology in the investigative sequence of hepatic lesions with an emphasis on hepatocellular carcinoma. J Cytol.

[12] Guo Z, Kurtycz DF, Salem R, De Las Casas LE, Caya JG, Daniel Hoerl H (2002). Radiologically guided percutaneous fine-needle aspiration biopsy of the liver: retrospective study of 119 cases evaluating diagnostic effectiveness and clinical complications. Diagn Cytopathol.

[13] Soyuer I, Ekinci C, Kaya M, Genç Y, Bahar K (2003). Diagnosis of hepatocellular carcinoma by fine needle aspiration cytology: cellular features. Acta Cytologica.

[14] Namshiker AA, Rocha P, Pinto R (2021). The utility of fine needle aspiration cytology in the assessment of hepatic lesions. NJLM.

[15] Zhang BC, Wang NJ, Huang XY, Wang MR, Zhang QM, Shao LQ (1986). Fine needle aspiration cytology in the diagnosis of primary liver cancer [Article in Chinese]. Zhonghua Zhong Liu Za Zhi.

[16] Xu GA (1989). Ultrasonically guided fine-needle biopsy of space-occupying lesions in liver [Article in Chinese]. Zhonghua Zhong Liu Za Zhi.

[17] Al-Damegh SA (2004). Ultrasound guided fine needle aspiration using 25G needle as a new technique for a wide range of pathological conditions. Saudi Med J.

[18] Lever JV, Trott PA, Webb AJ (1985). Fine needle aspiration cytology. J Clin Pathol.

[19] Rasania A, Pandey CL, Joshi N (2007). Evaluation of FNAC in diagnosis of hepatic lesion. J Cytol.

